# Low molecular weight carbohydrates and abiotic stress tolerance in lentil (*Lens culinaris* Medikus): a review

**DOI:** 10.3389/fpls.2024.1408252

**Published:** 2024-10-03

**Authors:** Mark Dempsey, Dil Thavarajah

**Affiliations:** Plant and Environmental Sciences, Pulse Quality and Nutritional Breeding, Biosystems Research Complex, Clemson University, Clemson, SC, United States

**Keywords:** pulse crops, biofortification, lentil, low molecular weight carbohydrates, raffinose family oligosaccharides, sugar alcohols, abiotic stress

## Abstract

Lentil (*Lens culinaris* Medikus) is a nutrient-rich, cool-season food legume that is high in protein, prebiotic carbohydrates, vitamins, and minerals. It is a staple food in many parts of the world, but crop performance is threatened by climate change, where increased temperatures and less predictable precipitation can reduce yield and nutritional quality. One mechanism that many plant species use to mitigate heat and drought stress is the production of disaccharides, oligosaccharides and sugar alcohols, collectively referred to as low molecular weight carbohydrates (LMWCs). Recent evidence indicates that lentil may also employ this mechanism – especially raffinose family oligosaccharides and sugar alcohols – and that these may be suitable targets for genomic-assisted breeding to improve crop tolerance to heat and drought stress. While the genes responsible for LMWC biosynthesis in lentil have not been fully elucidated, single nucleotide polymorphisms and putative genes underlying biosynthesis of LMWCs have been identified. Yet, more work is needed to confirm gene identity, function, and response to abiotic stress. This review i) summarizes the diverse evidence for how LMWCs are utilized to improve abiotic stress tolerance, ii) highlights current knowledge of genes that control LMWC biosynthesis in lentil, and iii) explores how LMWCs can be targeted using diverse genomic resources and markers to accelerate lentil breeding efforts for improved stress tolerance.

## 1 Introduction

Lentil (*Lens culinaris* Medikus) is a nutrient-rich, cool-season food legume produced in dry regions worldwide. Lentil has been cultivated for over 10,000 years and was domesticated in the fertile crescent (Cubero, 1981). Lentil is an herbaceous, self-pollinating diploid with seven chromosomes (2*n* = 14). Total global production has been increasing over the last two decades (Kaale et al., 2023), with recent production at 6.16 ± 0.47 million tons annually (5-year mean ± standard deviation: 2018-2022), concentrated in Canada (36% of global production), India (22%), Australia (11%), Turkey (6%), and the United States (4%) (FAO, 2024). Production is expected to continue rising in the coming decades to help feed the growing human population, as lentil is high in protein (20 – 25%), rich in carbohydrates (60 – 63%) and many micronutrients, and low in fat (1.5 – 3%) (Johnson et al., 2020; Sen Gupta et al., 2013; Tahir et al., 2011; Thavarajah et al., 2011; Wang and Daun, 2006; Zhang et al., 2014). Lentil seeds contain high concentrations of prebiotic carbohydrates (11 – 25%), which are not directly digested by humans but are fermented in the gastrointestinal tract by beneficial microorganisms, and are associated with a healthy gut microbiome and other health benefits (Beserra et al., 2015; Gibson et al., 2017; Johnson et al., 2021). Lentil prebiotic carbohydrates consist of three basic carbohydrate classes: oligosaccharides (0.9 – 6.1 g/100 g), sugar alcohols (SAs; 0.25 – 1.7 g/100 g), and resistant starch (3.7 – 22.1 g/100 g) (Johnson et al., 2015, 2021). There are two classes of oligosaccharides in lentil: raffinose family oligosaccharides (RFOs; 0.9 – 6.0 g/100 g) and fructooligosaccharides (FOS; 0.06 – 0.09 g/100 g), with RFOs constituting the vast majority (*ca*. 97 – 99%) of oligosaccharides in lentil (**Table 1**).

**Table 1:** Concentration ranges (mg/100 g) of LMWCs and broad sense heritability estimates (H^2^) in pulse crops.

Component	Lentil (H^2^)	Pea (H^2^)	Chickpea (H^2^)
	mg/100g		
Raffinose + Stachyose	578 – 3771(0.41)	2247 – 4813 (0.64)	1530 – 6840
Verbascose + Kestose	318 – 2253 (0.29)	1207 – 3078(0.45)	54 – 190
**Total RFOs**	**896 – 6025 (0.85)**	**3654 – 7890**	**2147 – 6973**
Nystose	48 – 62	1.6 – 9.1	NM
**Total Oligosaccharides**	**896 -6111**	**3654 – 7890**	**2147 – 6973**
Sorbitol	207 – 1496 (0.34)	8.4 – 115 (0.42)	NM
Mannitol	46 – 203 (0.45)	0.9 – 23.8 (0.57)	NM
Other SAs	46 – 89	192 – 856 (0.52 – 0.74)	331 – 2700
**Total SAs**	**253 – 1660**	**201 - 995**	**331 – 2700**
**Total LMWCs**	**1149 – 7772**	**3654 – 7890**	**2478 – 8980**
References	Johnson et al., 2013, 2015, 2021; Tahir et al., 2011	Gawłowska et al., 2022; Thavarajah et al., 2022; Vidal-Valverde et al., 2003	Alajaji and El-Adawy, 2006; Frias et al., 2000; Gangola et al., 2013; Rossi et al., 1984; Saini and Knights, 1984

NM, not measured.

Bold text indicates sums of LMWC groups where appropriate.

Oligosaccharides and SAs are critical throughout the lifecycle of many plant species, including legumes, as they improve tolerance to abiotic stress, such as high temperatures, drought, saline conditions, and oxidative stress (Benkeblia, 2022; Merchant and Richter, 2011; Yan et al., 2022). Sucrose and other disaccharides also improve tolerance to abiotic stress (Guy et al., 1992; Koster, 1991; Morelli et al., 2003). RFOs, FOS, SAs, and mono- and disaccharides are referred to in this review article as low molecular weight carbohydrates (LMWCs) and are defined in the following section. While the role of LMWCs in stress tolerance has not been explicitly studied in lentil, recent research showed LMWCs in lentil seeds varied significantly across nine environments, with the total LMWC concentration positively correlated with growing season temperature (Johnson et al., 2015). This research suggests that higher temperatures can lead to more LMWC accumulation in seeds, possibly in response to heat or water-deficit stress. Thus, because of its favorable nutrient profile and potential stress tolerance, lentil is a good candidate for adaptation to a changing climate.

Higher temperatures and frequent droughts will put unprecedented pressure on crop production systems within the next century (Coughlan de Perez et al., 2023; Heino et al., 2023; Robinson et al., 2021). This pressure will be intensified by the expanding nutritional needs of the global human population (UN-DESA, 2024). Many have called for wide-ranging efforts to meetthese increasing food and fiber needs without further degrading the environment (Foley et al., 2011; Godfray et al., 2010; Hunter et al., 2017). Understanding how staple crops such as lentil and other pulses cope with heat and drought stress is critical to ensure global food security. Given the importance of LWMCs for stress tolerance in crops, understanding the genetic underpinnings of LMWC biosynthesis is an essential step towards developing new climate change-resilient cultivars. However, the genetic basis of LMWC biosynthesis has not been well characterized in lentil, especially related to stress tolerance. A better understanding of the genetic basis of LMWC biosynthesis will enable breeders to make more targeted selections to hasten the release of stress-tolerant lentil cultivars. The objectives of this review are to i) describe the role of LMWCs in abiotic stress tolerance, ii) summarize current knowledge of the genes involved in LMWC biosynthesis, especially in response to abiotic stress, and iii) demonstrate how LMWCs can be targeted using diverse genomic resources and markers to accelerate lentil breeding efforts for improved stress tolerance.

## 2 Carbohydrates in plants

Plant carbohydrates are generally classified by the type, number, and linkage configuration of monosaccharides bonded to form more complex carbohydrates (**Table 2**). The monosaccharides glucose, fructose, and galactose are bonded in various configurations to form polymers of increasing complexity or are reduced to form SAs. Sucrose is a disaccharide consisting of one glucose molecule and one fructose molecule; it is the primary carbon transport molecule in plants, is used to synthesize many essential compounds, and helps to mitigate abiotic stress (Huber and Huber, 1996; Kühn et al., 1999; Nägele et al., 2012; Peshev et al., 2013). Other disaccharides such as trehalose and maltose are common in plants; trehalose metabolism is tightly linked with sucrose metabolism (Lunn et al., 2014), and maltose is a starch breakdown product that is important in many aspects of carbon metabolism (Fincher, 1989; Lu and Sharkey, 2006). Oligosaccharides are carbohydrates composed of three to 20 polymerized monosaccharides (Cummings and Stephen, 2007) and have diverse physiological roles in plants, such as carbon storage, stress tolerance, and carbon transport in certain taxa (Hannah et al., 2006; Yan et al., 2022). Among oligosaccharides, RFOs and FOS are the two most abundant classes in plants (Van den Ende, 2013). Sugar alcohols such as sorbitol and mannitol are derived from glucose or fructose by one or more chemical reduction steps and have many functions in plants, including carbon transport and storage and stress tolerance (Dumschott et al., 2017; Loescher and Everard, 2000). Starch is referred to as a high molecular weight carbohydrate and is comprised of two highly polymerized carbohydrates: amylopectin (70 – 85% of starch by weight; degree of polymerization *ca*. 40 – 50) and amylose (15 – 30% of starch; degree of polymerization *ca*. 30) (Cummings and Stephen, 2007). Starch is the primary form of carbon storage in plants (MacNeill et al., 2017). Resistant starch is a nutritional term referring to starch that is not readily digested because it is i) bound within a food matrix and physically inaccessible to enzyme activity, ii) inaccessible to enzyme activity due to granule type, especially when raw, iii) recrystallized after cooking and cooling (retrograded), iv) structurally modified, or v) complexed with a lipid (Cummings and Stephen, 2007; Dhital et al., 2016; Gutiérrez and Tovar, 2021).

**Table 2:** Classification of common carbohydrates in plants.

Low Molecular Weight Carbohydrates					High Molecular Weight Carbohydrates	
Mono-saccharides	Di-saccharides	Sugar Alcohols	Oligosaccharides		Starch	
			Raffinose Family Oligosaccharides	Fructo- oligosaccharides		
Glucose Fructose Galactose	Sucrose Trehalose Maltose	Sorbitol Mannitol Xylitol	Raffinose Stachyose Verbascose	Kestose Nystose	Resistant Starch	Digestible Starch

**Figure 1:**
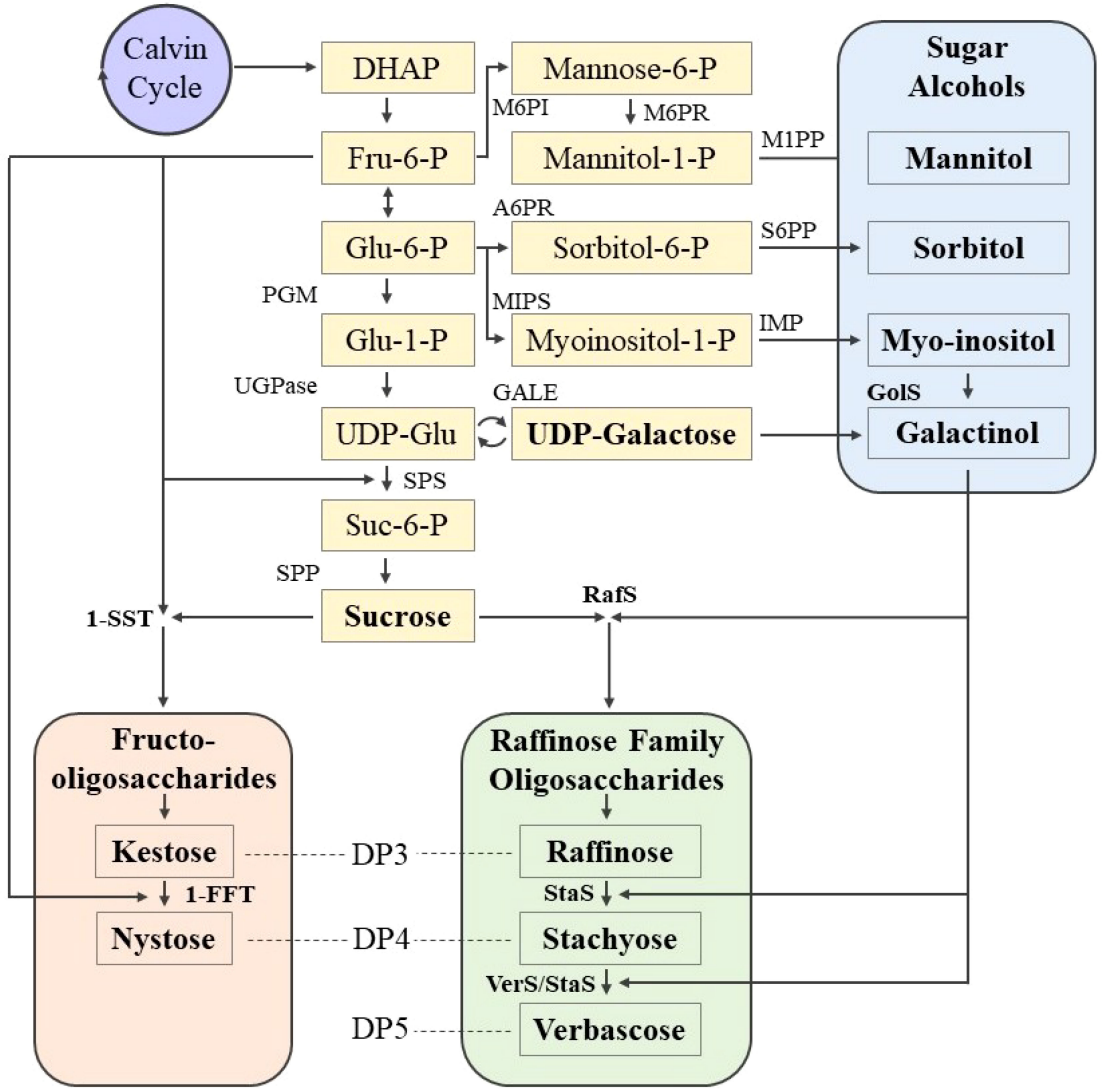
Biosynthetic pathways of key LMWCs involved in stress tolerance: RFOs, FOS, and SAs. Myo-inositol is a SA, although its critical role in stress tolerance appears to be related to galactinol formation. Bold text indicates LMWCs directly involved in stress response, their immediate precursors, and their key enzymes. Abbreviations: DP, degree of polymerization; DHAP, dihydroxyacetone phosphate; UDP, uridine diphosphate; Fru, fructose; Glu, glucose; Suc, sucrose; P, phosphate; PGM, phosphoglucomutase; UGPase, UDP-glucose-pyrophosphorylase; SPS, sucrose phosphate synthase; SPP, sucrose phosphatase; GolS, galactinol synthase; RafS, raffinose synthase; StaS, stachyose synthase; VerS, verbascose synthase; 1-SST, sucrose:sucrose 1-fructosyl-transferase; 1-FFT, fructan:fructan 1-fructosyltransferase; M6PI, mannose-6-phosphate isomerase; M6PR, mannose-6-phosphate reductase; M1PP, mannitol-1-phosphate phosphatase; A6PR, aldose-6-phosphate reductase; S6PP, sorbitol-6-phosphate phosphatase; MIPS, myoinositol-1-phosphate synthase; IMP, inositol mono phosphatase; GALE, UDP-galactose 4-epimerase and UDP-glucose 4-epimerase. Figure created from Dumschott et al. (2017); Johnson et al. (2020); Sanyal et al. (2023), and Singh et al. (2017).

The biosynthetic pathways of sucrose, oligosaccharides, and SAs have been elucidated in many plant species. These begin with fructose, glucose, or galactose, which are combined or modified to form a variety of more complex or reduced carbohydrates (**Figure 1**) (Dumschott et al., 2017; Johnson et al., 2020; Sanyal et al., 2023; Singh et al., 2017). Sucrose is synthesized from modified forms of glucose and fructose, i.e., uridine diphosphate glucose and fructose-6-phosphate, in a reaction catalyzed by sucrose phosphate synthase to form sucrose-6-phosphate, which is then converted to sucrose by sucrose phosphatase (Winter and Huber, 2000). The biosynthesis of RFOs typically begins with the formation of galactinol from myo-inositol and uridine diphosphate-galactose, the galactose donor, and is catalyzed by galactinol synthase (*GolS* or *GS*) (Peterbauer and Richter, 2001). Raffinose is formed from sucrose, and the galactosyl is transferred from galactinol, catalyzed by raffinose synthase (*RafS* or *RS*). Stachyose is formed from raffinose and galactinol (galactosyl donor), and is catalyzed by stachyose synthase (*StaS* or *STS*). Verbascose is formed from stachyose and galactinol, and may be catalyzed by verbascose synthase or stachyose synthase (Elango et al., 2022; Lahuta et al., 2010). Biosynthesis of FOS begins with the formation of kestose from sucrose and fructose, and is catalyzed by sucrose:sucrose 1-fructosyl-transferase. Nystose is formed from kestose and fructose, catalyzed by fructan:fructan 1-fructosyltransferase (Singh et al., 2017; Vijn and Smeekens, 1999). SA biosynthesis begins, generally, with either fructose or glucose. Mannitol is formed from fructose-6-phosphate, with two intermediate forms (mannose-6-phosphate and mannitol-1-phosphate), catalyzed by mannose-6-phosphate isomerase, mannose-6-phosphate reductase, and mannitol-1-phosphate phosphatase. Sorbitol is formed from glucose-6-phosphate, with sorbitol-6-phosphate as an intermediate step and is catalyzed by aldose-6-phosphate reductase and sorbitol-6-phosphate phosphatase (Dumschott et al., 2017; Loescher and Everard, 2000). Biosynthetic pathways for LMWCs have not yet been elucidated in lentil because these pathways are common among most flowering plants (Benkeblia, 2022; Loescher and Everard, 2000; Salerno and Curatti, 2003; Sengupta et al., 2015).

## 3 Low molecular weight carbohydrates and abiotic stress

**Figure 2:**
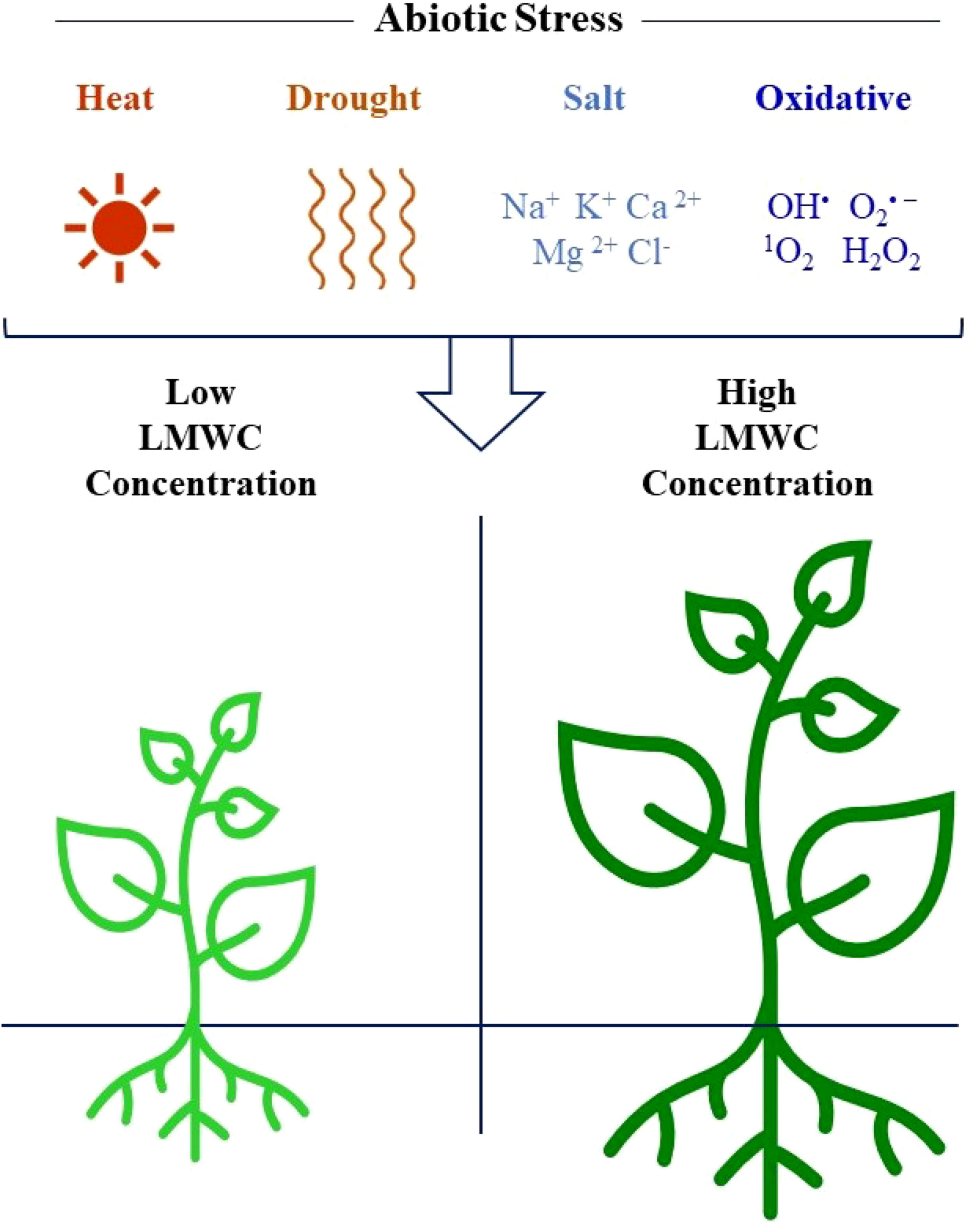
Conceptual diagram of the diverse roles of LMWCs in abiotic stress tolerance in plants.

Among LMWCs, oligosaccharides and SAs are emerging as key compounds that plants use to manage abiotic stresses, such as extreme temperatures, drought, salinity, and oxidative damage (Cacela and Hincha, 2006; Corbineau et al., 2000; Shimosaka and Ozawa, 2015; Stoyanova et al., 2011) (**Figure 2**). The main oligosaccharides produced by lentil are RFOs, with only small amounts of FOS produced by these crops (Johnson et al., 2015, 2021) (**Table 1**).

### 3.1 Osmo-protection

Water deficit stress can occur when plants are subjected to high heat, drought, or saline conditions, negatively affecting plant growth and ultimately leading to reduced crop yield and nutritional content (Barnabás et al., 2008; Choukri et al., 2020; Sehgal et al., 2018). Plants have evolved complex physiological and biochemical mechanisms to ameliorate water deficit stress, including stomatal closure, lower photosynthesis rates, and accumulation of small organic molecules in cells that maintain membrane integrity and osmotic pressure, among many others (Gururani et al., 2015; Hincha et al., 2003; Liang et al., 2020).

The potential osmo-protective role of disaccharides and oligosaccharides has been studied *in vitro* using a model membrane system (Crowe et al., 1984; Hincha et al., 2003). These studies demonstrate that sucrose, trehalose, and several oligosaccharides can reduce membrane desiccation damage by two potential mechanisms (Hincha et al., 2003; Koster, 1991). First, the water replacement hypothesis proposes that hydroxyl groups on LMWCs form hydrogen bonds with lipid headgroups in membranes, stabilizing membranes during dehydration and minimizing leakage (Hincha et al., 2003). Second, the cytoplasmic vitrification hypothesis proposes that the accumulation of LMWCs in cells leads to the formation of “sugar glass,” which immobilizes membranes and cytoplasmic macromolecules, protecting them from damage (Cacela and Hincha, 2006; Koster, 1991).

LMWC accumulation in response to water deficit has been observed in many plant species, including lentil. Disaccharide and oligosaccharide accumulation has been observed during drought conditions in the seeds of maize (*Zea mays*; Koster and Leopold, 1988; Mohammadkhani and Heidari, 2008), soybean (*Glycine max*; Blackman et al., 1992), field pea (*Pisum sativum*; Corbineau et al., 2000), and beech (*Fagus sylvatica*; Pukacka et al., 2009), and is thought to confer desiccation tolerance to seeds (Corbineau et al., 2000; Koster and Leopold, 1988). Drought conditions have also led to disaccharide or oligosaccharide accumulation in the stems or leaves of lentil (Foti et al., 2021), wheat (*Triticum aestivum*; Hou et al., 2018; Zhang et al., 2015) and chickpea (*Cicer arietinum*; Salvi et al., 2018), as well as the roots of chicory (*Cichorium intybus*; De Roover et al., 2000) and *Vernonia herbacea* (Garcia et al., 2011), suggesting a critical role of disaccharides and oligosaccharides in managing drought stress in vegetative tissues. The accumulation of SAs in vegetative tissues in response to drought or salt stress has also been observed in the leaves of soybean (Streeter et al., 2001), rice bean (*Vigna umbellata*; Wanek et al., 1997), chickpea (Orthen et al., 2000), and kiwi (*Actinidia deliciosa*; Klages et al., 1999). In lentil, several studies have linked drought and/or heat stress with higher concentrations of LMWCs. Foti et al. (2021) found that drought stress increased the concentrations of the disaccharide α,α-trehalose and D-myo-inositol phosphate (an RFO precursor) in a drought-tolerant genotype. Other studies have found that total LMWC or RFO concentrations in lentil seeds were linked to high temperatures and/or low precipitation, possibly in response to heat or drought stress (Johnson et al., 2015a; Graham et al., 2017). These insights point to the need for targeted research into the potential osmo-protective role of LMWCs in lentil to inform future breeding efforts.

### 3.2 Antioxidants

Another role of LMWCs in abiotic stress tolerance is reducing oxidative damage. Reactive oxygen species (ROS) are byproducts of plant metabolism that can damage proteins, lipids, and nucleic acids at high concentrations. These damaging compounds primarily consist of hydroxyl radicals (OH^•^), superoxide ion radicals (O_2_^• –^), singlet oxygen (^1^O_2_), and hydrogen peroxide (H_2_O_2_; Peshev and Van Den Ende, 2013). Plants employ various antioxidant mechanisms to scavenge ROS: vitamins C and E, enzyme-based systems such as catalase superoxidase dismutase (among others), and several secondary metabolites such as carotenoids, flavonoids, and terpenoids (Gill and Tuteja, 2010). Under optimal conditions, ROS are scavenged at the same rate they are produced by plant metabolic processes. During stress, however, the ability of plants to use these antioxidant mechanisms is diminished, leading to oxidative damage. Recent research demonstrates the role of several LMWCs in scavenging ROS to limit oxidative damage (Matros et al., 2015; Van Den Ende and Valluru, 2009). Many plant-derived disaccharides (e.g., sucrose, trehalose, and maltose), RFOs, and FOS scavenge ROS with varying degrees of affinity. In general, monosaccharides such as glucose and fructose are not effective ROS scavengers compared to disaccharides, oligosaccharides, or SAs (Morelli et al., 2003; Peshev et al., 2013; Stoyanova et al., 2011). Peshev et al. (2013) showed a 10-fold difference in ROS-scavenging capacity between trehalose (lowest capacity among carbohydrates tested) and inulin (a FOS; highest capacity). In Matros et al. (2015), *A. thaliana* plants were supplied with sucralose, a synthetic sucrose analog, and demonstrated a carbohydrate-antioxidant mechanism that decreased oxidative stress induced by paraquat and UV-B. While this experimental work is currently limited to *A. thaliana* and select vegetable crops, the scavenging of ROS by LMWCs may extend to other plant species, including lentil.

## 4 Gene identification and function: biosynthesis of low molecular weight carbohydrates

Much research has been carried out to identify the genes responsible for the biosynthesis of LMWCs in response to abiotic stress. To achieve this, studies have inserted, eliminated, or modified genes responsible for RFO, FOS, and SA biosynthetic pathways in many plant species, with corresponding changes in LMWC concentrations and abiotic stress tolerance (Bie et al., 2012; Egert et al., 2013; Panikulangara et al., 2004; Zhifang and Loescher, 2003).

### 4.1 Oligosaccharides

A critical step in the biosynthesis of RFOs is the formation of galactinol from myo-inositol and UDP-galactose, which is catalyzed by the enzyme *GolS*. Thus, manipulating *GolS* genes can affect downstream RFO concentrations and abiotic stress tolerance. For example, Panikulangara et al. (2004) found that upregulating *GolS* genes in *A. thaliana* increased raffinose concentrations in leaves and improved tolerance to heat and drought stress. In contrast, eliminating *GolS* genes had the opposite effect on raffinose and stress tolerance. Another study overexpressed *GolS* genes from chickpea (*CaGolS*1 and *CaGolS*2) in *A. thaliana*, which led to higher RFO concentrations in vegetative tissues and fewer signs of stress in the face of elevated temperatures (Salvi et al., 2018). Nishizawa et al. (2008) used a different approach to increase RFOs via *GolS*, where they overexpressed heat shock transcription factor A2 (HsfA2) to induce the transcription of *GolS* in *A. thaliana.* This led to increased raffinose concentrations in leaves and reduced oxidative damage. Putative *GolS* genes have also been identified in lentil (*LcGolS1* and *LcGolS2*) using a cDNA library prepared from developing seeds, where nucleotide sequences were aligned from *Medicago sativa*, field pea, soybean, and *Ammopiptanthus mongolicus* (Kannan et al., 2016). However, follow-up work is needed to confirm these genes and their role in RFO biosynthesis in response to abiotic stress. For example, a transformation system could be employed to overexpress and suppress putative *LcGolS* genes, followed by quantification of gene expression, galactinol and RFOs to confirm gene identity. Similarly, the role of RFOs in abiotic stress tolerance in lentil could be further elucidated by exposing transformed plants to abiotic stress, and any differences in RFO concentrations and crop performance between transformed genotypes (overexpressing vs. suppressing *LcGolS*) would provide information about gene function in response to abiotic stress.

The next step in the biosynthetic pathway of RFOs is raffinose synthesis, catalyzed by *RafS*. Functional studies of *RafS* genes in many plant species have confirmed the important role of RFOs in abiotic stress tolerance (Li et al., 2020). Egert et al. (2013) demonstrated in *A. thaliana* using two loss-of-function mutants that a raffinose synthase gene (*AtRafS5*) is solely responsible for raffinose accumulation in seeds and leaves in response to drought, salinity, and oxidative stress. Li et al. (2020) demonstrated a raffinose synthase gene from maize (*ZmRafS*) is induced by drought, heat and salinity stress, and that a maize mutant lacking *ZmRafS* is drought sensitive compared to a maize null-segregant with this gene. They also found overexpression of *ZmRafS* in *A. thaliana* resulted in enhanced drought tolerance and increased raffinose concentrations in seeds. Similar to putative *GolS* genes in lentil (Kannan et al., 2016), putative genes for *RafS* and *StaS* identified using cDNA library have also been identified in lentil (Kannan et al., 2021); significant follow-up work is needed to confirm the presence of these genes and their function when faced with abiotic stress.

Fructooligosaccharides also have a role in stress tolerance. Transgenic studies show exotic genes responsible for FOS biosynthesis from bacteria (*Bacillus subtilis*) and several plant species (wheat, Jerusalem artichoke (*Helianthus tuberosus)*, onion (*Allium cepa)*, and *Psathyrostachys huashanica*) result in increased FOS concentrations and improved drought tolerance in tobacco (Bie et al., 2012; He et al., 2015; Pilon-Smits et al., 1995; Sun et al., 2020), sugar beet (Pilon-Smits et al., 1999), and cotton (Liu et al., 2022). Fructooligosaccharide concentrations in lentil seeds are considerably lower than RFO concentrations (*ca.* 1 – 3% of total oligosaccharides; Johnson et al., 2015), and therefore are likely less important for stress tolerance compared to RFOs.

These studies have identified the genes responsible for oligosaccharide biosynthesis in many plant species and suggest *GolS* and *RafS* genes are good candidates for increasing RFOs in lentil in order to improve tolerance to abiotic stress (Egert et al., 2013; Ma et al., 2021; Salvi et al., 2018). Given that putative genes for *GolS* and *RafS* have been identified in lentil, with better-described *GolS* and *RafS* genes in pea and chickpea (Lahuta et al., 2014; Salvi et al., 2018), further research is clearly needed to confirm the identity and function of *GolS* and *RafS* genes in lentil to support breeding efforts targeting abiotic stress tolerance. Genes encoding enzymes involved in the biosynthesis of RFOs in lentil and other plant species are shown in **Table 3**.

**Table 3:** Genes encoding enzymes involved in the biosynthesis of LMWCs in lentil and other plants.

Carbohydrates		Enzyme Involved in Carbohydrate Biosynthesis	Genes		
			Lentil	Other Species	References
RFOs & Precursors	Myo-inositol	Inositol mono phosphatase (IMP; EC 3.1.3.25)	LcIMP (putative)	CaIMP (*C. arietinum*); AtIMPL & AtVTC4 (*A. thaliana*)	Torabinejad et al., 2009; Sato et al., 2011; Saxena et al., 2013; Song et al., 2022
	Galactinol	Galactinol synthase (GolS or GS; EC 2.4.1.123)	LcGolS (putative)	PsGolS(*P. sativum*); CaGolS (*C. arietinum*); PvGS (*Phaseolus vulgaris*); AtGolS(*A. thaliana*)	Liu et al., 1998; Taji et al., 2002; Lahuta et al., 2014; Kannan et al., 2016; Salvi et al., 2018
	Raffinose	Raffinose synthase (RafS or RS; EC 2.4.1.82)	LcRafS (putative)	PsRS (*P. sativum*); AtRS(*A. thaliana*)	Peterbauer et al., 2002a; Egert et al., 2013; Kannan et al., 2021
	Stachyose	Stachyose synthase (StaS or STS; EC 2.4.1.67)	LcSTS (putative)	PsSTS (*P. sativum*); AtSTS (*A. thaliana*; putative)	Peterbauer et al., 2002b; Gangl et al., 2015; Kannan et al., 2021
	Verbascose	Verbascose synthase (VerS)	StaS is likely responsible for verbascose biosynthesis (*P. sativum* & *L. culinaris*)		Kannan et al., 2021; Peterbauer et al., 2002b
SAs	Mannitol	Mannose-6-phosphate reductase (M6PR; EC 1.1.1.224)	NI	AgM6PR (*A. graveolens*); CaM6PR (*Coffea arabica*)	Everard et al., 1997; de Carvalho et al., 2014
	Sorbitol	Aldose-6-phosphate reductase (A6PR; EC 1.1.1.200)	NI	MdA6PR (*Malus domestica*)	Meng et al., 2023

NI, indicates that genes have not been identified for specific enzymes.

### 4.2 Sugar alcohols

The role of SAs in abiotic stress mitigation has not been studied as well as that of other LMWCs, but several transgenic studies demonstrate the important role of SAs in stress tolerance. For example, genes responsible for mannitol biosynthesis (mannitol-1-phosphate dehydrogenase) transgenically introduced from *E. coli* improved drought and salinity stress in basmati rice (*Oryza sativa*; Pujni et al., 2007), and mannose-6-phosphate reductase genes introduced from celery (*Apium graveolens*) improved tolerance to salinity stress in *A. thaliana* (Zhifang and Loescher, 2003). Transgenes responsible for D-ononitol biosynthesis in a salt-tolerant rice were introduced to tobacco and increased both D-ononitol and tolerance to abiotic stresses (drought and salinity) (Sheveleva et al., 1997). Similarly, genes responsible for myo-inositol biosynthesis were introduced to tobacco, leading to increased tolerance to salinity stress (Majee et al., 2004).

Genes responsible for SA biosynthesis in lentil have not been characterized, and further research is required to understand the potential genetic underpinnings of SA biosynthesis in response to stress. Because the major SAs in lentil seeds are sorbitol and mannitol (*ca*. 86% and 13%, respectively; Johnson et al., 2013, 2015, 2021), research should focus on enzymes within these presumed biosynthetic pathways in lentil (**Figure 1**). Further, aldose-6-phosphate reductase (A6PR) and sorbitol-6-phosphate phosphatase (S6PP) may be ideal starting points, given sorbitol is considerably higher than mannitol in lentil (**Table 1**). Genes that encode for enzymes involved in sorbitol biosynthesis in plants are understudied, with the exception of Rosaceous tree fruits, and need further clarification in lentil and other annual crop species (**Table 3**).

## 5 Breeding potential, targets, and future directions

LMWCs in lentil and other pulse crops have been studied for crop improvement (Johnson et al., 2021; Thavarajah et al., 2022). Within the last decade, several studies have confirmed the genetic basis for variation of RFOs and SAs, suggesting these LMWCs in lentil can be increased with targeted breeding (Johnson et al., 2015, 2021). Broad-sense heritability estimates (H^2^) for LMWCs in lentil have not been well defined, but Johnson et al. (2021) found values ranged from 0.29 – 0.41 for RFOs and 0.34 – 0.45 for SAs within a diverse population of 143 lentil accessions (**Table 1**). These values are similar to those reported for other pulse crops: values for RFOs ranged from 0.25 – 0.56 in chickpea (Gangola et al., 2013) and 0.44 – 0.54 in common bean (McPhee et al., 2002). Similarly, Thavarajah et al. (2022) studied the heritability of LMWCs in field peas and found H^2^ values ranging from 0.64 – 0.74 for RFOs and 0.42 – 0.66 for SAs. Such moderate heritability values for LMWCs in lentil and related crops are likely due to the quantitative nature of these diverse traits. These results suggest conventional breeding efforts should be paired with genomic approaches to improve selection accuracy and efficiency to accelerate the development of new stress-tolerant lentil cultivars.

**Figure 3:**
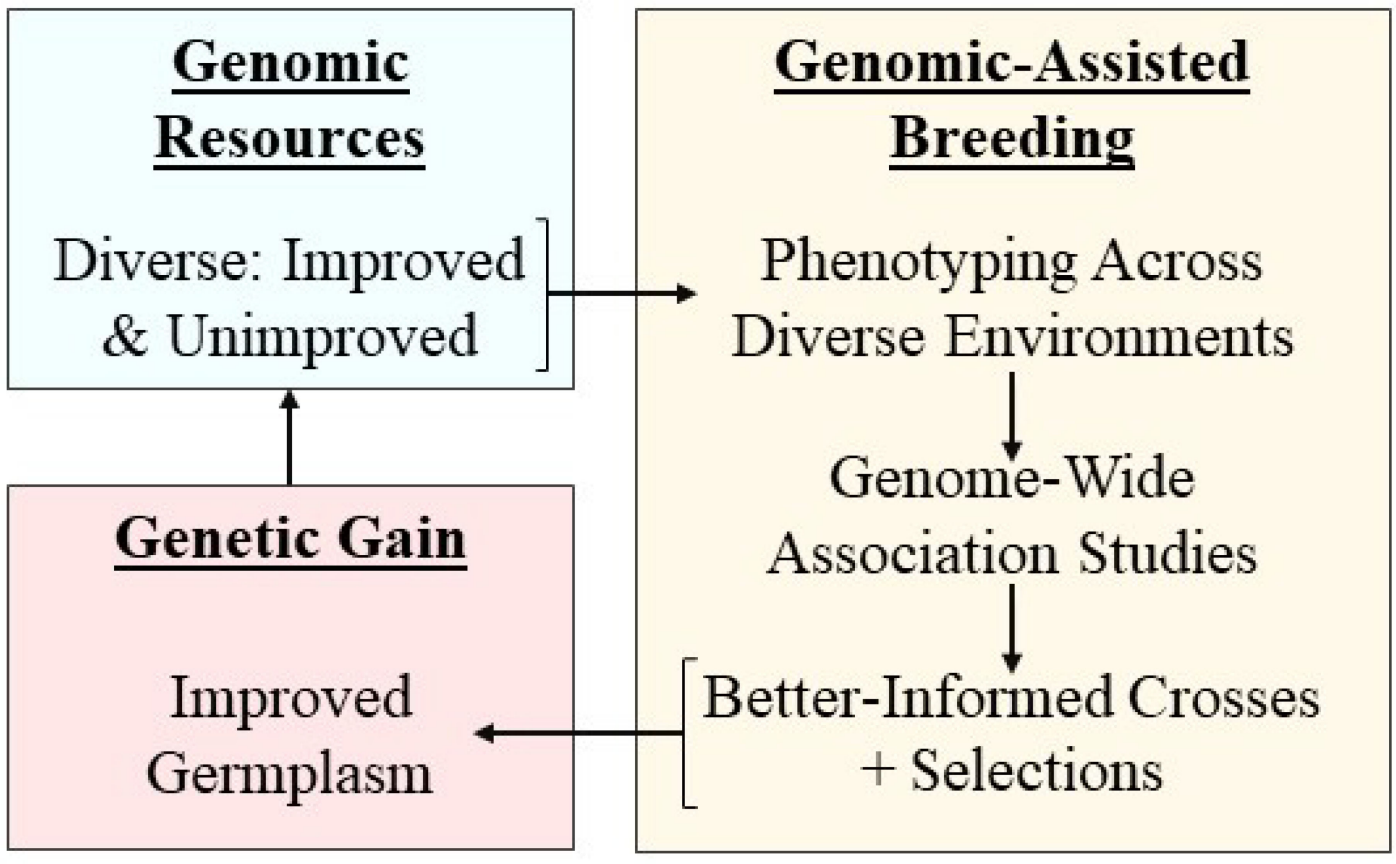
Simplified genomic-assisted breeding schematic, where diverse genomic resources are phenotyped across diverse environments, followed by association studies to correlate traits with genomic regions, which can inform parent selection for crosses, leading to improved germplasm.

Modern genomic-assisted breeding has improved the quality and quantity of genetic data available to plant breeders to develop more climate change-resilient cultivars. This is a powerful approach because it allows the selection of parents based on higher-resolution genomic data and sophisticated statistical techniques that identify genomic regions associated with desired traits. For example, genome-wide association studies (GWAS) can aid in the identification of genes by first identifying single nucleotide polymorphisms (SNPs) and quantitative trait loci (QTL) associated with specific traits. Confirmed genes, SNPs, and QTL can then be used to improve the accuracy of selecting breeding parents by avoiding selection based solely on phenotypic information. Genomic-assisted breeding is especially useful for complex, quantitative traits that are influenced by environmental factors (Kumar et al., 2016). Thus, breeding programs that utilize genomic-assisted techniques such as GWAS can make more targeted crosses and increase genetic gain more quickly than conventional breeding programs (**Figure 3**).

While the genomic regions responsible for the biosynthesis of LWMCs in lentil have received limited study, important progress toward gene identification has been made (Johnson et al., 2021; Kannan et al., 2016, 2021). Specifically, Kannan et al. (2016, 2021) identified putative *GolS*, *RafS*, and *StaS* genes from a cDNA library, and SNPs for mannitol and the sum of raffinose and stachyose have been identified using genome-wide association mapping (Johnson et al., 2021). These findings hold promise for continued elucidation of the genetic basis of LMWC biosynthesis in lentil, especially as more diverse genomic resources are characterized. As genomic resources for lentil are built, increased diversity will improve the predictive ability of statistical techniques used to identify candidate genes, and the diversity of potential parents will improve as well. Once QTL or genes involved in LMWC biosynthesis are better understood in lentil, more research will be required to confirm their function. For example, up- or downregulating *GolS* and *RafS* genes in lentil will help to confirm gene function, as determined by differences in RFO concentrations. Further, abiotic stress studies should be conducted in coordination with gene studies to improve our understanding of gene function and the concomitant role of LMWCs in stress tolerance in lentil. Specifically, testing crop performance as abiotic stresses are applied to lentil genotypes with up- or downregulating *GolS* and *RafS* genes will help to elucidate gene identify and function.

Within the context of a genomic-assisted breeding program, target LMWC concentrations should be developed. Target LMWC concentrations in lentil should consider their potential benefits for both human and plant health, as well as the potential drawbacks of consuming large amounts of oligosaccharides and SAs, which can lead to gastrointestinal distress in certain populations or individuals (Douglas and Sanders, 2008). While concentrations have not been established to maximize benefits for human or plant health, mean RFO (6.11 g/100 g) and SA (1.68 g/100 g) concentrations of nine commercial cultivars field-grown in six countries were below the recommended daily allowance (RDA) values of 7-30 g/day suggested for oligosaccharides (Coussement, 1999; Johnson et al., 2013; Silk et al., 2009). For sensitive individuals, these concentrations may lead to gastrointestinal discomfort but are at the low end of suggested RDA values and will likely not negatively affect most individuals.

Concentrations for LMWCs such as RFOs and SAs in vegetative tissue, which are important to consider given their critical role throughout the crop’s life cycle, are not often measured because most research has focused on the nutrient content and digestibility of lentil seeds. Thus, target concentrations for LMWCs in lentil vegetative tissues will require further study to identify optimal concentrations under different environmental conditions, especially abiotic stress. Any physiological tradeoffs between LMWC biosynthesis and other aspects of carbon metabolism should also be considered when determining target LMWC concentrations.

To make efficient progress toward new stress-tolerant lentil cultivars, future work must rely on diverse genomic resources and recent advances in genomic techniques such as whole-genome sequencing and complimentary statistical analyses. Yet, more research is needed to better understand the genes responsible for LMWC biosynthesis in lentil, as well optimal quantities for both human and plant health. With this information, genomic-assisted breeding techniques can be employed to hasten the development of stress tolerant lentil cultivars for a more food secure future.

## 6 Conclusion

Lentil is a nutrient-dense food crop that is well adapted to the challenging growing conditions of the dry regions where it is primarily produced, and it is well positioned to contribute to global food security in the future. However, more research is needed to take better advantage of LMWCs in lentil to improve tolerance to heat and drought stress, thereby addressing the dual threats posed by climate change and a growing human population. Importantly, LMWCs in lentil also provide health benefits to consumers, which is significant for all global consumers. Broadening and characterizing lentil genomic resources are the first steps toward better understanding the genes responsible for LMWC biosynthesis in lentil, followed by confirming gene function as stress response compounds. From this work, diverse genomic resources and genomic-assisted breeding techniques can be leveraged to develop more climate-resilient lentil cultivars based on LMWCs. Thus, by employing genomic-assisted breeding techniques that focus on LMWCs, lentil yield and nutritional quality can be maintained or improved, helping to ensure food security in a changing world.

## References

[B1] Alajaji S. A. El-Adawy T. A. (2006). Nutritional composition of chickpea (*Cicer arietinum* L.) as affected by microwave cooking and other traditional cooking methods. J. Food Composition Anal. 19, 806–812. doi: 10.1016/J.JFCA.2006.03.015

[B2] Barnabás B. Jäger K. Fehér A. (2008). The effect of drought and heat stress on reproductive processes in cereals. Plant Cell Environ. 31, 11–38. doi: 10.1111/J.1365-3040.2007.01727.X 17971069

[B3] Benkeblia N. (2022). Insights on fructans and resistance of plants to drought stress. Front. Sustain Food Syst. 6. doi: 10.3389/fsufs.2022.827758

[B4] Beserra B. T. S. Fernandes R. do Rosario V. A. Mocellin M. C. Kuntz M. G. F. Trindade E. B. S. M. (2015). A systematic review and meta-analysis of the prebiotics and synbiotics effects on glycaemia, insulin concentrations and lipid parameters in adult patients with overweight or obesity. Clin. Nutr. 34, 845–858. doi: 10.1016/J.CLNU.2014.10.004 25456608

[B5] Bie X. Wang K. She M. Du L. Zhang S. Li J.. (2012). Combinational transformation of three wheat genes encoding fructan biosynthesis enzymes confers increased fructan content and tolerance to abiotic stresses in tobacco. Plant Cell Rep. 31, 2229–2238. doi: 10.1007/S00299-012-1332-Y 22911265

[B6] Blackman S. A. Obendorf R. L. Leopold A. C. (1992). Maturation proteins and sugars in desiccation tolerance of developing soybean seeds. Plant Physiol. 100, 225. doi: 10.1104/PP.100.1.225 16652951 PMC1075542

[B7] Cacela C. Hincha D. K. (2006). Monosaccharide composition, chain length and linkage type influence the interactions of oligosaccharides with dry phosphatidylcholine membranes. Biochim. Biophys. Acta 1758, 680–691. doi: 10.1016/J.BBAMEM.2006.04.005 16730644

[B8] Choukri H. Hejjaoui K. El-Baouchi A. El haddad N. Smouni A. Maalouf F.. (2020). Heat and drought stress impact on phenology, grain yield, and nutritional quality of lentil (*Lens culinaris* medikus). Front. Nutr. 7. doi: 10.3389/FNUT.2020.596307 PMC7719779 33330596

[B9] Corbineau F. Picard M. A. Fougereux J. A. Ladonne F. Côme D. (2000). Effects of dehydration conditions on desiccation tolerance of developing pea seeds as related to oligosaccharide content and cell membrane properties. Seed Sci. Res. 10, 329–339. doi: 10.1017/S0960258500000374

[B10] Coughlan de Perez E. Ganapathi H. Masukwedza G. I. T. Griffin T. Kelder T. (2023). Potential for surprising heat and drought events in wheat-producing regions of USA and China. NPJ Climate Atmospheric Sci. 6, 1–10. doi: 10.1038/s41612-023-00361-y PMC11041665 38665270

[B11] Coussement P. A. A. (1999). Inulin and oligofructose: safe intakes and legal status. J. Nutr. 129, 1412S–1417S. doi: 10.1093/JN/129.7.1412S 10395609

[B12] Crowe L. M. Mouradian R. Crowe J. H. Jackson S. A. Womersley C. (1984). Effects of carbohydrates on membrane stability at low water activities. Biochim. Biophys. Acta (BBA) - Biomembranes 769, 141–150. doi: 10.1016/0005-2736(84)90017-8 6691971

[B13] Cubero J. I. (1981). “Origin, domestication and evolution,” in Lentils, eds. Webb C. Hawtin G. C. (Slough, UK: Commonwealth of Agriculture Bureau), 15–38.

[B14] Cummings J. H. Stephen A. M. (2007). Carbohydrate terminology and classification. Eur. J. Clin. Nutr. 61, S5–S18. doi: 10.1038/sj.ejcn.1602936 17992187

[B15] de Carvalho K. Petkowicz C. L. O. Nagashima G. T. Bespalhok Filho J. C. Vieira L. G. E. Pereira L. F. P.. (2014). Homeologous genes involved in mannitol synthesis reveal unequal contributions in response to abiotic stress in *Coffea arabica* . Mol. Genet. Genomics 289, 951–963. doi: 10.1007/S00438-014-0864-Y 24861101

[B16] De Roover J. Vandenbranden K. Van Laere A. Van Den Ende W. (2000). Drought induces fructan synthesis and 1-SST (sucrose:sucrose fructosyltransferase) in roots and leaves of chicory seedlings (*Cichorium intybus* L.). Planta 210, 808–814. doi: 10.1007/S004250050683 10805453

[B17] Dhital S. Warren F. J. Butterworth P. J. Ellis P. R. Gidley M. J. (2016). Mechanisms of starch digestion by α-amylase—Structural basis for kinetic properties. Crit. Rev. Food Sci. Nutr. 57, 875–892. doi: 10.1080/10408398.2014.922043 25751598

[B18] Douglas L. C. Sanders M. E. (2008). Probiotics and prebiotics in dietetics practice. J. Am. Diet Assoc. 108, 510–521. doi: 10.1016/J.JADA.2007.12.009 18313433

[B19] Dumschott K. Richter A. Loescher W. Merchant A. (2017). Post photosynthetic carbon partitioning to sugar alcohols and consequences for plant growth. Phytochemistry 144, 243–252. doi: 10.1016/J.PHYTOCHEM.2017.09.019 28985572

[B20] Egert A. Keller F. Peters S. (2013). Abiotic stress-induced accumulation of raffinose in *Arabidopsis* leaves is mediated by a single raffinose synthase (RS5, At5g40390). BMC Plant Biol. 13, 1–9. doi: 10.1186/1471-2229-13-218 24354450 PMC3878221

[B21] Elango D. Rajendran K. van der Laan L. Sebastiar S. Raigne J. Thaiparambil N. A.. (2022). Raffinose family oligosaccharides: friend or foe for human and plant health? Front. Plant Sci. 13. doi: 10.3389/FPLS.2022.829118 PMC8891438 35251100

[B22] Everard J. D. Cantini C. Grumet R. Plummer J. Loescher W. H. (1997). Molecular cloning of mannose-6-phosphate reductase and its developmental expression in celery. Plant Physiol. 113, 1427–1435. doi: 10.1104/pp.113.4.1427 9112783 PMC158267

[B23] FAO. (2024). FAOSTAT: Crops and livestock products. Available online at: https://www.fao.org/faostat/en/#data/QCL (Accessed July, 31, 2024).

[B24] Fincher G. B. (1989). Molecular and cellular biology associated with endosperm mobilization in germinating cereal grains. Annu. Rev. Plant Physiol. Plant Mol. Bioi 40, 305–351. doi: 10.1146/annurev.pp.40.060189.001513

[B25] Foley J. A. Ramankutty N. Brauman K. A. Cassidy E. S. Gerber J. S. Johnston M.. (2011). Solutions for a cultivated planet. Nature 478, 337–342. doi: 10.1038/nature10452 21993620

[B26] Foti C. Kalampokis I. F. Aliferis K. A. Pavli O. I. (2021). Metabolic responses of two contrasting lentil genotypes to PEG-induced drought stress. Agronomy 11, 1190. doi: 10.3390/AGRONOMY11061190

[B27] Frias J. Vidal-Valverde C. Sotomayor C. Diaz-Pollan C. Urbano G. (2000). Influence of processing on available carbohydrate content and antinutritional factors of chickpeas. Eur. Food Res. Technol. 210, 340–345. doi: 10.1007/S002170050560

[B28] Gangl R. Behmüller R. Tenhaken R. (2015). Molecular cloning of AtRS4, a seed specific multifunctional RFO synthase/galactosylhydrolase in *Arabidopsis thaliana* . Front. Plant Sci. 6. doi: 10.3389/FPLS.2015.00789 PMC4587089 26483807

[B29] Gangola M. P. Khedikar Y. P. Gaur P. M. Baìšga M. Chibbar R. N. (2013). Genotype and growing environment interaction shows a positive correlation between substrates of raffinose family oligosaccharides (RFO) biosynthesis and their accumulation in chickpea (*Cicer arietinum* L.) seeds. J. Agric. Food Chem. 61, 4943–4952. doi: 10.1021/JF3054033/SUPPL_FILE/JF3054033_SI_001.PDF 23621405

[B30] Garcia P. M. A. Asega A. F. Silva E. A. Carvalho M. A. M. (2011). Effect of drought and re-watering on fructan metabolism in *Vernonia herbacea* (Vell.) Rusby. Plant Physiol. Biochem. 49, 664–670. doi: 10.1016/J.PLAPHY.2011.03.014 21531568

[B31] Gawłowska M. Lahuta L. Boros L. Sawikowska A. Kumar P. Knopkiewicz M.. (2022). Validation of Molecular markers significant for flowering time, plant lodging, stem geometry properties, and raffinose family oligosaccharides in pea (*Pisum sativum* L.). Agric. (Switzerland) 12, 1125. doi: 10.3390/AGRICULTURE12081125/S1

[B32] Gibson G. R. Hutkins R. Sanders M. E. Prescott S. L. Reimer R. A. Salminen S. J.. (2017). Expert consensus document: The International Scientific Association for Probiotics and Prebiotics (ISAPP) consensus statement on the definition and scope of prebiotics. Nat. Rev. Gastroenterol. Hepatol. 14, 491–502. doi: 10.1038/nrgastro.2017.75 28611480

[B33] Gill S. S. Tuteja N. (2010). Reactive oxygen species and antioxidant machinery in abiotic stress tolerance in crop plants. Plant Physiol. Biochem. 48, 909–930. doi: 10.1016/J.PLAPHY.2010.08.016 20870416

[B34] Godfray H. C. J. Beddington J. R. Crute I. R. Haddad L. Lawrence D. Muir J. F.. (2010). Food security: the challenge of feeding 9 billion people. Science (1979) 327, 812–818. doi: 10.1126/science.1185383 20110467

[B35] Graham C. Thavarajah D. Beck R. (2017). Carbohydrate concentration in lentils (*Lens culinaris* medikus.): genotypic and environmental effects. Comm Soil Sci. Plant Anal. 48, 2447–2454. doi: 10.1080/00103624.2017.1411937

[B36] Gururani M. A. Venkatesh J. Tran L. S. P. (2015). Regulation of photosynthesis during abiotic stress-induced photoinhibition. Mol. Plant 8, 1304–1320. doi: 10.1016/J.MOLP.2015.05.005 25997389

[B37] Gutiérrez T. J. Tovar J. (2021). Update of the concept of type 5 resistant starch (RS5): Self-assembled starch V-type complexes. Trends Food Sci. Technol. 109, 711–724. doi: 10.1016/J.TIFS.2021.01.078

[B38] Guy C. L. Huber J. L. A. Huber S. C. (1992). Sucrose phosphate synthase and sucrose accumulation at low temperature. Plant Physiol. 100, 502–508. doi: 10.1104/PP.100.1.502 16652990 PMC1075578

[B39] Hannah M. A. Zuther E. Buchel K. Heyer A. G. (2006). Transport and metabolism of raffinose family oligosaccharides in transgenic potato. J. Exp. Bot. 57, 3801–3811. doi: 10.1093/JXB/ERL152 17050641

[B40] He X. Chen Z. Wang J. Li W. Zhao J. Wu J.. (2015). A sucrose:fructan-6-fructosyltransferase (6-SFT) gene from *Psathyrostachys huashanica* confers abiotic stress tolerance in tobacco. Gene 570, 239–247. doi: 10.1016/J.GENE.2015.06.023 26072162

[B41] Heino M. Kinnunen P. Anderson W. Ray D. K. Puma M. J. Varis O.. (2023). Increased probability of hot and dry weather extremes during the growing season threatens global crop yields. Sci. Rep. 13, 1–13. doi: 10.1038/s41598-023-29378-2 36869041 PMC9984494

[B42] Hincha D. K. Zuther E. Heyer A. G. (2003). The preservation of liposomes by raffinose family oligosaccharides during drying is mediated by effects on fusion and lipid phase transitions. Biochim. Biophys. Acta (BBA) - Biomembranes 1612, 172–177. doi: 10.1016/S0005-2736(03)00116-0 12787935

[B43] Hou J. Huang X. Sun W. Du C. Wang C. Xie Y.. (2018). Accumulation of water-soluble carbohydrates and gene expression in wheat stems correlates with drought resistance. J. Plant Physiol. 231, 182–191. doi: 10.1016/J.JPLPH.2018.09.017 30278314

[B44] Huber S. C. Huber J. L. (1996). Role and regulation of sucrose-phosphate synthase in higher plants. Annu. Rev. Plant Physiol. Plant Mol. Biol. 47, 431–444. doi: 10.1146/ANNUREV.ARPLANT.47.1.431 15012296

[B45] Hunter M. C. Smith R. G. Schipanski M. E. Atwood L. W. Mortensen D. A. (2017). Agriculture in 2050: recalibrating targets for sustainable intensification. Bioscience 67, 386–391. doi: 10.1093/BIOSCI/BIX010

[B46] Johnson N. Boatwright J. L. Bridges W. Thavarajah P. Kumar S. Shipe E.. (2021). Genome-wide association mapping of lentil (*Lens culinaris* Medikus) prebiotic carbohydrates toward improved human health and crop stress tolerance. Sci. Rep. 11, 1–12. doi: 10.1038/s41598-021-93475-3 34230595 PMC8260633

[B47] Johnson N. Johnson C. R. Thavarajah P. Kumar S. Thavarajah D. (2020). The roles and potential of lentil prebiotic carbohydrates in human and plant health. Plants People Planet 2, 310–319. doi: 10.1002/PPP3.10103

[B48] Johnson C. R. Thavarajah D. Combs G. F. Thavarajah P. (2013). Lentil (*Lens culinaris* L.): A prebiotic-rich whole food legume. Food Res. Int. 51, 107–113. doi: 10.1016/J.FOODRES.2012.11.025

[B49] Johnson C. R. Thavarajah D. Thavarajah P. Fenlason A. McGee R. Kumar S.. (2015). A global survey of low-molecular weight carbohydrates in lentils. J. Food Composition Anal. 44, 178–185. doi: 10.1016/J.JFCA.2015.08.005

[B50] Kaale L. D. Siddiq M. Hooper S. (2023). Lentil (*Lens culinaris* Medik) as nutrient-rich and versatile food legume: A review. Legume Sci. 5, e169. doi: 10.1002/LEG3.169

[B51] Kannan U. Sharma R. GAngola M. P. Ganeshan S. Båga M. Chibbar R. N. (2021). Sequential expression of raffinose synthase and stachyose synthase corresponds to successive accumulation of raffinose, stachyose and verbascose in developing seeds of Lens culinaris Medik. J. Plant Physiol. 265, 153494. doi: 10.1016/J.JPLPH.2021.153494 34454370

[B52] Kannan U. Sharma R. Khedikar Y. GAngola M. P. Ganeshan S. Båga M.. (2016). Differential expression of two galactinol synthase isoforms LcGolS1 and LcGolS2 in developing lentil (*Lens culinaris* Medik. cv CDC Redberry) seeds. Plant Physiol. Biochem. 108, 422–433. doi: 10.1016/J.PLAPHY.2016.08.004 27552180

[B53] Klages K. Boldingh H. Smith G. S. (1999). Accumulation of myo -inositol in *actinidia* seedlings subjected to salt stress. Ann. Bot. 84, 521–527. doi: 10.1006/ANBO.1999.0946

[B54] Koster K. L. (1991). Glass formation and desiccation tolerance in seeds. Plant Physiol. 96, 302. doi: 10.1104/PP.96.1.302 16668169 PMC1080750

[B55] Koster K. L. Leopold A. C. (1988). Sugars and desiccation tolerance in seeds. Plant Physiol. 88, 829. doi: 10.1104/PP.88.3.829 16666392 PMC1055669

[B56] Kühn C. Barker L. Bürkle L. Frommer W. (1999). Update on sucrose transport in higher plants. J. Exp. Bot. 50, 935–953. doi: 10.1093/jxb/50.Special_Issue.935

[B57] Kumar J. Sen Gupta D. Kumar S. Gupta S. Singh N. P. (2016). Current knowledge on genetic biofortification in lentil. J. Agric. Food Chem. 64, 6383–6396. doi: 10.1021/ACS.JAFC.6B02171 27507630

[B58] Lahuta L. B. Goszczynska J. Horbowicz M. (2010). Seed alpha-D-galactosides of selected Vicia species and enzymes involved in their biosynthesis. Acta Biol. Crac Ser. Bot. 52, 27–35.

[B59] Lahuta L. B. Pluskota W. E. Stelmaszewska J. Szablińska J. (2014). Dehydration induces expression of Galactinol Synthase And Raffinose Synthase in seedlings of pea (*Pisum sativum* L.). J. Plant Physiol. 171, 1306–1314. doi: 10.1016/J.JPLPH.2014.04.012 25014266

[B60] Li T. Zhang Y. Liu Y. Li X. Hao G. Han Q.. (2020). Raffinose synthase enhances drought tolerance through raffinose synthesis or galactinol hydrolysis in maize and *Arabidopsis* plants. J. Biol. Chem. 295, 8064–8077. doi: 10.1074/JBC.RA120.013948 32366461 PMC7278351

[B61] Liang G. Liu J. Zhang J. Guo J. (2020). Effects of drought stress on photosynthetic and physiological parameters of tomato. J. Am. Soc. Hortic. Sci. 145, 12–17. doi: 10.21273/JASHS04725-19

[B62] Liu R. N. Jiao T. Q. Zhang Z. X. Yao Z. Li Z. Q. Wang S.. (2022). Ectopic expression of the allium cepa 1-SST gene in cotton improves drought tolerance and yield under drought stress in the field. Front. Plant Sci. 12. doi: 10.3389/FPLS.2021.783134 PMC8790044 35095957

[B63] Liu J. J. J. Krenz D. C. Galvez A. F. De Lumen B. O. (1998). Galactinol synthase (GS): increased enzyme activity and levels of mRNA due to cold and desiccation. Plant Sci. 134, 11–20. doi: 10.1016/S0168-9452(98)00042-9

[B64] Loescher W. H. Everard J. D. (2000). “Regulation of Sugar Alcohol Biosynthesis,” in Photosynthesis: Physiology and Metabolism, eds Leegood R. Sharkey T. D. Caemmerer S. (Dordrecht, Netherlands: Springer) 9, 275–299.

[B65] Lu Y. Sharkey T. D. (2006). The importance of maltose in transitory starch breakdown. Plant Cell Environ. 29, 353–366. doi: 10.1111/J.1365-3040.2005.01480.X 17080591

[B66] Lunn J. E. Delorge I. Figueroa C. M. Van Dijck P. Stitt M. (2014). Trehalose metabolism in plants. Plant J. 79, 544–567. doi: 10.1111/TPJ.12509 24645920

[B67] Ma S. Lv J. Li X. Ji T. Zhang Z. Gao L. (2021). Galactinol synthase gene 4 (CsGolS4) increases cold and drought tolerance in *Cucumis sativus* L. by inducing RFO accumulation and ROS scavenging. Environ. Exp. Bot. 185, 104406. doi: 10.1016/J.ENVEXPBOT.2021.104406

[B68] MacNeill G. J. Mehrpouyan S. Minow M. A. A. Patterson J. A. Tetlow I. J. Emes M. J. (2017). Starch as a source, starch as a sink: the bifunctional role of starch in carbon allocation. J. Exp. Bot. 68, 4433–4453. doi: 10.1093/JXB/ERX291 28981786

[B69] Majee M. Maitra S. Dastidar K. G. Pattnaik S. Chatterjee A. Hait N. C.. (2004). A Novel Salt-tolerant l-myo-Inositol-1-phosphate Synthase from *Porteresia coarctata* (Roxb.) Tateoka, a Halophytic Wild Rice: Molecular Cloning, Bacterial Overexpression, Characterization, and Functional Introgression into Tobacco-Conferring Salt Tolerance Phenotype. J. Biol. Chem. 279, 28539–28552. doi: 10.1074/JBC.M310138200 15016817

[B70] Matros A. Peshev D. Peukert M. Mock H. P. Van Den Ende W. (2015). Sugars as hydroxyl radical scavengers: proof-of-concept by studying the fate of sucralose in Arabidopsis. Plant J. 82, 822–839. doi: 10.1111/TPJ.12853 25891826

[B71] McPhee K. E. Zemetra R. S. Brown J. Myers J. R. (2002). Genetic analysis of the raffinose family oligosaccharides in common bean. J. Am. Soc. Hortic. Sci. 127, 376–382. doi: 10.21273/JASHS.127.3.376

[B72] Meng D. Cao H. Yang Q. Zhang M. Borejsza-Wysocka E. Wang H.. (2023). SnRK1 kinase-mediated phosphorylation of transcription factor bZIP39 regulates sorbitol metabolism in apple. Plant Physiol. 192, 2123–2142. doi: 10.1093/PLPHYS/KIAD226 37067900 PMC10315300

[B73] Merchant A. Richter A. A. (2011). Polyols as biomarkers and bioindicators for 21st century plant breeding. Funct. Plant Biol. 38, 934–940. doi: 10.1071/FP11105 32480952

[B74] Mohammadkhani N. Heidari R. (2008). Drought-induced accumulation of soluble sugars and proline in two maize varieties. World Appl. Sci. J. 3, 448–453.

[B75] Morelli R. Russo-Volpe S. Bruno N. Lo Scalzo R. (2003). Fenton-dependent damage to carbohydrates: free radical scavenging activity of some simple sugars. J. Agric. Food Chem. 51, 7418–7425. doi: 10.1021/JF030172Q 14640593

[B76] Nägele T. Stutz S. Hörmiller I. I. Heyer A. G. (2012). Identification of a metabolic bottleneck for cold acclimation in *Arabidopsis thaliana* . Plant J. 72, 102–114. doi: 10.1111/J.1365-313X.2012.05064.X 22640594

[B77] Nishizawa A. Yabuta Y. Shigeoka S. (2008). Galactinol and raffinose constitute a novel function to protect plants from oxidative damage. Plant Physiol. 147, 1251–1263. doi: 10.1104/PP.108.122465 18502973 PMC2442551

[B78] Orthen B. Popp M. Barz W. (2000). Cyclitol accumulation in suspended cells and intact plants of *Cicer arietinum* L. J. Plant Physiol. 156, 40–45. doi: 10.1016/S0176-1617(00)80270-9

[B79] Panikulangara T. J. Eggers-Schumacher G. Wunderlich M. Stransky H. Schöffl F. (2004). Galactinol synthase1. A novel heat shock factor target gene responsible for heat-induced synthesis of raffinose family oligosaccharides in *arabidopsis* . Plant Physiol. 136, 3148. doi: 10.1104/PP.104.042606 15466240 PMC523375

[B80] Peshev D. Van Den Ende W. (2013). “Sugars as Antioxidants in Plants,” in Crop Improvement Under Adverse Conditions, eds Tuteja N. Singh Gill S. (New York, United States: Springer), 285–307. doi: 10.1007/978-1-4614-4633-0_13

[B81] Peshev D. Vergauwen R. Moglia A. Hideg É. Van Den Ende W. (2013). Towards understanding vacuolar antioxidant mechanisms: a role for fructans? J. Exp. Bot. 64, 1025–1038. doi: 10.1093/JXB/ERS377 23349141 PMC3580814

[B82] Peterbauer T. Mach L. Mucha J. Richter A. (2002a). Functional expression of a cDNA encoding pea (Pisum sativum L.) raffinose synthase, partial purification of the enzyme from maturing seeds, and steady-state kinetic analysis of raffinose synthesis. Planta 215, 839–846. doi: 10.1007/S00425-002-0804-7 12244450

[B83] Peterbauer T. Mucha J. Mach L. Richter A. (2002b). Chain Elongation of raffinose in pea seeds. Isolation, characterization, and molecular cloning of mutifunctional enzyme catalyzing the synthesis of stachyose and verbascose. J. Biol. Chem. 277, 194–200. doi: 10.1074/JBC.M109734200 11675396

[B84] Peterbauer T. Richter A. (2001). Biochemistry and physiology of raffinose family oligosaccharides and galactosyl cyclitols in seeds. Seed Sci. Res. 11, 185–197. doi: 10.1079/SSR200175

[B85] Pilon-Smits E. A. H. Ebskamp M. J. M. Paul M. J. Jeuken M. J. W. Weisbeek P. J. Smeekens S. C. M. (1995). Improved performance of transgenic fructan-accumulating tobacco under drought stress. Plant Physiol. 107, 125–130. doi: 10.1104/PP.107.1.125 12228347 PMC161174

[B86] Pilon-Smits E. A. H. Terry N. Sears T. Van Dun K. (1999). Enhanced drought resistance in fructan-producing sugar beet. Plant Physiol. Biochem. 37, 313–317. doi: 10.1016/S0981-9428(99)80030-8

[B87] Pujni D. Chaudhary A. Rajam M. V. (2007). Increased tolerance to salinity and drought in transgenic indica rice by mannitol accumulation. J. Plant Biochem. Biotechnol. 16, 1–7. doi: 10.1007/BF03321921

[B88] Pukacka S. Ratajczak E. Kalemba E. (2009). Non-reducing sugar levels in beech (Fagus sylvatica) seeds as related to withstanding desiccation and storage. J. Plant Physiol. 166, 1381–1390. doi: 10.1016/J.JPLPH.2009.02.013 19359065

[B89] Robinson A. Lehmann J. Barriopedro D. Rahmstorf S. Coumou D. (2021). Increasing heat and rainfall extremes now far outside the historical climate. NPJ Climate Atmospheric Sci. 2021 4:1 4, 1–4. doi: 10.1038/s41612-021-00202-w

[B90] Rossi M. Germondari I. Casini P. (1984). Comparison of chickpea cultivars: chemical composition, nutritional evaluation, and oligosaccharide content. J. Agric. Food Chem. 32, 811–814. doi: 10.1021/JF00124A028/ASSET/JF00124A028

[B91] Saini H. S. Knights E. J. (1984). Chemical constitution of starch and oligosaccharide components of “desi” and “kabuli” Chickpea (*Cicer arietinum*) seed types. J. Agric. Food Chem. 32, 940–944. doi: 10.1021/JF00124A059/ASSET/JF00124A059

[B92] Salerno G. L. Curatti L. (2003). Origin of sucrose metabolism in higher plants: when, how and why? Trends Plant Sci. 8, 63. doi: 10.1016/S1360-1385(02)00029-8 12597872

[B93] Salvi P. Kamble N. U. Majee M. (2018). Stress-inducible galactinol synthase of chickpea (CaGolS) is implicated in heat and oxidative stress tolerance through reducing stress-induced excessive reactive oxygen species accumulation. Plant Cell Physiol. 59, 155–166. doi: 10.1093/PCP/PCX170 29121266

[B94] Sanyal R. Kumar S. Pattanayak A. Kar A. Bishi S. K. (2023). Optimizing raffinose family oligosaccharides content in plants: A tightrope walk. Front. Plant Sci. 14. doi: 10.3389/FPLS.2023.1134754 PMC10088399 37056499

[B95] Sato Y. Yazawa K. Yoshida S. Tamaoki M. Nakajima N. Iwai H.. (2011). Expression and functions of myo-inositol monophosphatase family genes in seed development of *Arabidopsis* . J. Plant Res. 124, 385–394. doi: 10.1007/S10265-010-0381-Y 20960216

[B96] Saxena S. C. Salvi P. Kaur H. Verma P. Petla B. P. Rao V.. (2013). Differentially expressed myo-inositol monophosphatase gene (CaIMP) in chickpea (*Cicer arietinum* L.) encodes a lithium-sensitive phosphatase enzyme with broad substrate specificity and improves seed germination and seedling growth under abiotic stresses. J. Exp. Bot. 64, 5623–5639. doi: 10.1093/JXB/ERT336 24123252 PMC3871819

[B97] Sehgal A. Sita K. Siddique K. H. M. Kumar R. Bhogireddy S. Varshney R. K.. (2018). Drought or/and heat-stress effects on seed filling in food crops: Impacts on functional biochemistry, seed yields, and nutritional quality. Front. Plant Sci. 871. doi: 10.3389/FPLS.2018.01705 PMC6277783 30542357

[B98] Sengupta S. Mukherjee S. Basak P. Majumder A. L. (2015). Significance of galactinol and raffinose family oligosaccharide synthesis in plants. Front. Plant Sci. 6. doi: 10.3389/FPLS.2015.00656 PMC4549555 26379684

[B99] Sen Gupta D. Thavarajah D. Knutson P. Thavarajah P. McGee R. J. Coyne C. J.. (2013). Lentils (*Lens culinaris* L.), a rich source of folates. J. Agric. Food Chem. 61, 7794–7799. doi: 10.1021/JF401891P 23865478

[B100] Sheveleva E. Chmara W. Bohnert H. J. Jensen R. G. (1997). Increased salt and drought tolerance by D-ononitol production in transgenic nicotiana tabacum L. Plant Physiol. 115, 1211–1219. doi: 10.1104/PP.115.3.1211 12223867 PMC158586

[B101] Shimosaka E. Ozawa K. (2015). Overexpression of cold-inducible wheat galactinol synthase confers tolerance to chilling stress in transgenic rice. Breed Sci. 65, 363. doi: 10.1270/JSBBS.65.363 26719738 PMC4671696

[B102] Silk D. B. A. Davis A. Vulevic J. Tzortzis G. Gibson G. R. (2009). Clinical trial: the effects of a trans-galactooligosaccharide prebiotic on faecal microbiota and symptoms in irritable bowel syndrome. Aliment Pharmacol. Ther. 29, 508–518. doi: 10.1111/J.1365-2036.2008.03911.X 19053980

[B103] Singh S. P. Jadaun J. S. Narnoliya L. K. Pandey A. (2017). Prebiotic oligosaccharides: special focus on fructooligosaccharides, its biosynthesis and bioactivity. Appl. Biochem. Biotechnol. 2017 183:2 183, 613–635. doi: 10.1007/S12010-017-2605-2 28948462

[B104] Song J. Mavraganis I. Shen W. Yang H. Cram D. Xiang D.. (2022). Transcriptome dissection of candidate genes associated with lentil seed quality traits. Plant Biol. 24, 815–826. doi: 10.1111/PLB.13426 35395134

[B105] Stoyanova S. Geuns J. Hideg É. Van Den Ende W. (2011). The food additives inulin and stevioside counteract oxidative stress. Int. J. Food Sci. Nutr. 62, 207–214. doi: 10.3109/09637486.2010.523416 21043580

[B106] Streeter J. G. Lohnes D. G. Fioritto R. J. (2001). Patterns of pinitol accumulation in soybean plants and relationships to drought tolerance. Plant Cell Environ. 24, 429–438. doi: 10.1046/J.1365-3040.2001.00690.X

[B107] Sun X. Zong Y. Yang S. Wang L. Gao J. Wang Y.. (2020). A fructan: The fructan 1-fructosyl-transferase gene from *Helianthus tuberosus* increased the PEG-simulated drought stress tolerance of tobacco. Hereditas 157, 1–8. doi: 10.1186/S41065-020-00131-3 32312318 PMC7171796

[B108] Tahir M. Lindeboom N. Båga M. Vandenberg A. Chibbar R. N. (2011). Composition and correlation between major seed constituents in selected lentil (Lens culinaris. Medik) genotypes. Can. J. Plant Sci. 91, 825–835. doi: 10.4141/CJPS2011-010

[B109] Taji T. Ohsumi C. Iuchi S. Seki M. Kasuga M. Kobayashi M.. (2002). Important roles of drought- and cold-inducible genes for galactinol synthase in stress tolerance in Arabidopsis thaliana. Plant J. 29, 417–426. doi: 10.1046/J.0960-7412.2001.01227.X 11846875

[B110] Thavarajah D. Lawrenceid T. J. Powers S. E. Kayid J. Thavarajah P. Shipe E.. (2022). Organic dry pea (*Pisum sativum* L.) biofortification for better human health (A.N. Shahzad, editor). PloS One 17, e0261109. doi: 10.1371/JOURNAL.PONE.0261109 35025919 PMC8757916

[B111] Thavarajah D. Thavarajah P. Wejesuriya A. Rutzke M. Glahn R. P. Combs G. F. Jr.. (2011). The potential of lentil (*Lens culinaris* L.) as a whole food for increased selenium, iron, and zinc intake: Preliminary results from a 3 year study. Euphytica 180, 123–128. doi: 10.1007/S10681-011-0365-6

[B112] Torabinejad J. Donahue J. L. Gunesekera B. N. Allen-Daniels M. J. Gillaspy G. E. (2009). VTC4 is a bifunctional enzyme that affects myoinositol and ascorbate biosynthesis in plants. Plant Physiol. 150, 951–961. doi: 10.1104/PP.108.135129 19339506 PMC2689953

[B113] UN-DESA (United Nations Department of Economic and Social Affairs, Population Division). (2024). World Population Prospects 2024: Summary of Results (UN DESA/POP/2024/TR/NO.9). Available online at: https://population.un.org/wpp/Publications/ (Accessed Sept. 24, 2024).

[B114] Van den Ende W. (2013). Multifunctional fructans and raffinose family oligosaccharides. Front. Plant Sci. 4. doi: 10.3389/FPLS.2013.00247 PMC3713406 23882273

[B115] Van Den Ende W. Valluru R. (2009). Sucrose, sucrosyl oligosaccharides, and oxidative stress: scavenging and salvaging? J. Exp. Bot. 60, 9–18. doi: 10.1093/JXB/ERN297 19036839

[B116] Vidal-Valverde C. Frias J. Hernández A. Martín-Alvarez P. J. Sierra I. Rodríguez C.. (2003). Assessment of nutritional compounds and antinutritional factors in pea (*Pisum sativum*) seeds. Sci. Food Ag 83, 298–306. doi: 10.1002/jsfa.1309

[B117] Vijn I. Smeekens S. (1999). Fructan: more than a reserve carbohydrate? Plant Physiol. 120, 351–360. doi: 10.1104/PP.120.2.351 10364386 PMC1539216

[B118] Wanek W. Richter Wanek A. Wanek W. Richter A. (1997). Biosynthesis and accumulation of D-ononitol in *Vigna umbellata* in response to drought stress. Physiol. Plant 101, 416–424. doi: 10.1111/J.1399-3054.1997.TB01016.X

[B119] Wang N. Daun J. K. (2006). Effects of variety and crude protein content on nutrients and anti-nutrients in lentils (*Lens culinaris*). Food Chem. 95, 493–502. doi: 10.1016/J.FOODCHEM.2005.02.001

[B120] Winter H. Huber S. C. (2000). Regulation of sucrose metabolism in higher plants: localization and regulation of activity of key enzymes. Crit. Rev. Biochem. Mol. Biol. 35, 253–289. doi: 10.1080/10409230008984165 11005202

[B121] Yan S. Liu Q. Li W. Yan J. Fernie A. R. (2022). Raffinose family oligosaccharides: crucial regulators of plant development and stress responses. CRC Crit. Rev. Plant Sci. 41, 286–303. doi: 10.1080/07352689.2022.2111756

[B122] Zhang J. Chen W. Dell B. Vergauwen R. Zhang X. Mayer J. E.. (2015). Wheat genotypic variation in dynamic fluxes of WSC components in different stem segments under drought during grain filling. Front. Plant Sci. 6. doi: 10.3389/FPLS.2015.00624 PMC4531436 26322065

[B123] Zhang B. Deng Z. Tang Y. Chen P. Liu R. Ramdath D. D.. (2014). Fatty acid, carotenoid and tocopherol compositions of 20 Canadian lentil cultivars and synergistic contribution to antioxidant activities. Food Chem. 161, 296–304. doi: 10.1016/J.FOODCHEM.2014.04.014 24837953

[B124] Zhifang G. Loescher W. H. (2003). Expression of a celery mannose 6-phosphate reductase in Arabidopsis thaliana enhances salt tolerance and induces biosynthesis of both mannitol and a glucosyl-mannitol dimer. Plant Cell Environ. 26, 275–283. doi: 10.1046/J.1365-3040.2003.00958.X

